# Congenital Hemangioma: A Case Report of a Finding Every Physician Should Know

**DOI:** 10.7759/cureus.2485

**Published:** 2018-04-16

**Authors:** Kamleshun Ramphul, Stephanie G Mejias, Yogeshwaree Ramphul-Sicharam, Ruhi Sonaye

**Affiliations:** 1 Department of Pediatrics, Shanghai Xin Hua Hospital Affiliated to Shanghai Jiao Tong University School of Medicine, Shanghai, CHN; 2 Department of Pediatrics, Robert Reid Cabral Children's Hospital Affiliated to the University Iberoamericana Unibe School of Medicine; 3 Sir Seewoosagur Ramgoolam National Hospital; 4 Bharati Vidyapeeth Deemed University Medical College and Hospital, Sangli, Maharashtra, IND

**Keywords:** congenital hemangiomas, pediatrics, vascular tumor

## Abstract

Congenital hemangiomas (CHs) are described as vascular tumors that appear as grown masses at birth. Most of the CHs are benign and they are divided into rapidly involuting congenital hemangiomas (RICHs), which usually resolve by the age of 14 months, and non-involuting congenital hemangiomas (NICHs), which persist and grows with age. There are multiple different conditions that may resemble the presentation of hemangiomas, and it is important to have an early differential diagnosis and tests to provide appropriate care. This case is about a newborn from Mauritius presenting with three vascular tumors diagnosed as congenital hemangiomas.

## Introduction

Congenital hemangiomas (CHs) are benign vascular tumors present at birth as fully growth masses. They usually present with exophytic masses or plaques on different parts of the body such as the head, limbs, or neck. They have been classified as rapidly involuting congenital hemangiomas (RICHs) and noninvoluting congenital hemangiomas (NICHs) [1]. RICHs usually resolve by the age of 14 months while the NICHs continue to grow and often need to be surgically excised. Retrospective studies have shown that 0.3% of newborns suffer from congenital hemangiomas [2], but its prevalence in Mauritius still remains unknown. This case involves a newborn with multiple congenital hemangiomas observed in Mauritius.

## Case presentation

A two-week-old female was brought in with an initial complaint of multiple masses that failed to regress since birth. She was born from a nonconsanguineous union and the mother’s pregnancy was uneventful. The baby was delivered vaginally at 37 weeks of gestation and multiples masses were found over the body. The treating physician advised follow-ups on discharge. However, the parents decided to seek more medical help for the child and she was admitted for more investigations.

**Figure 1:**
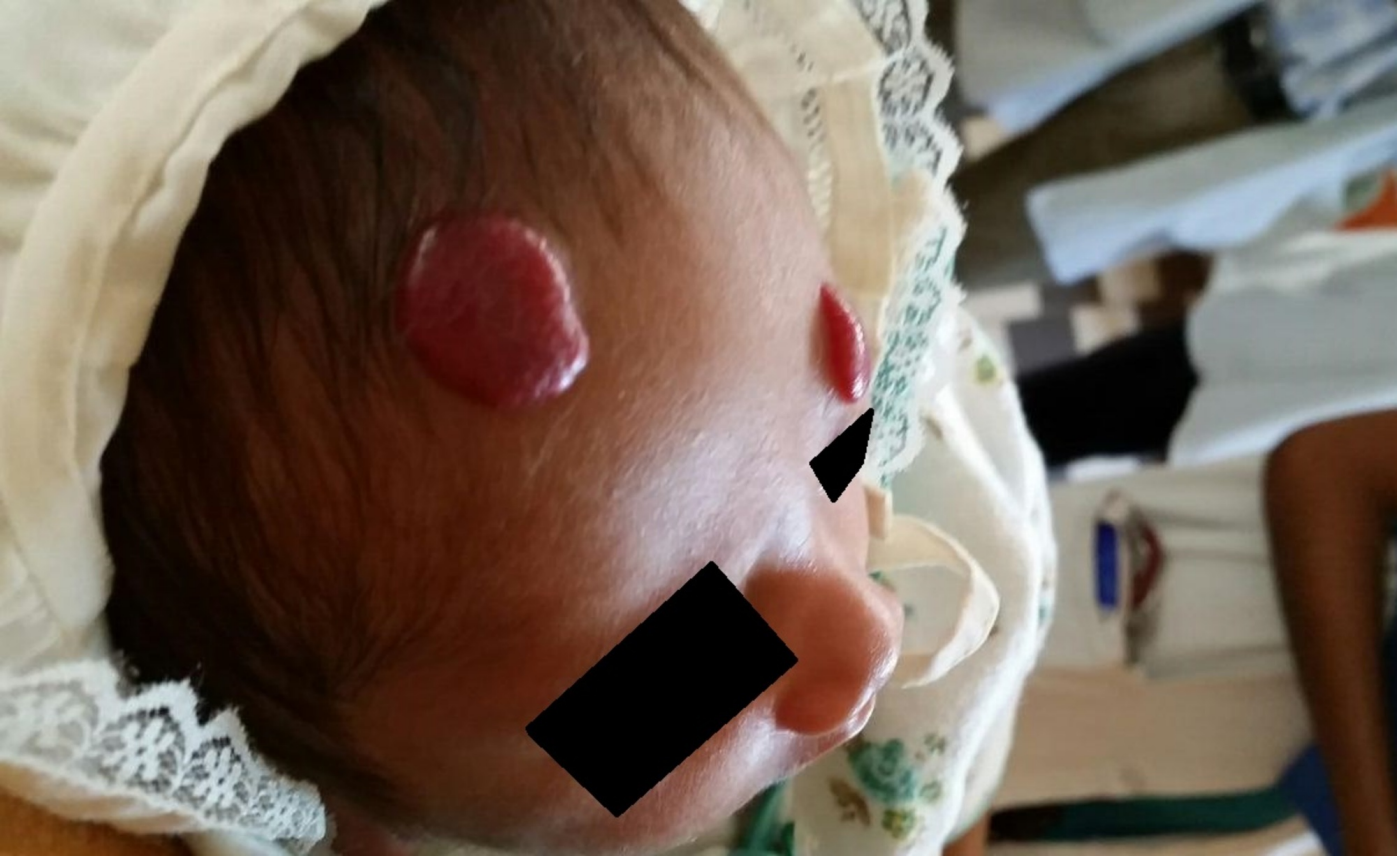
Two-week-old newborn with two hemangiomas on the head.

**Figure 2:**
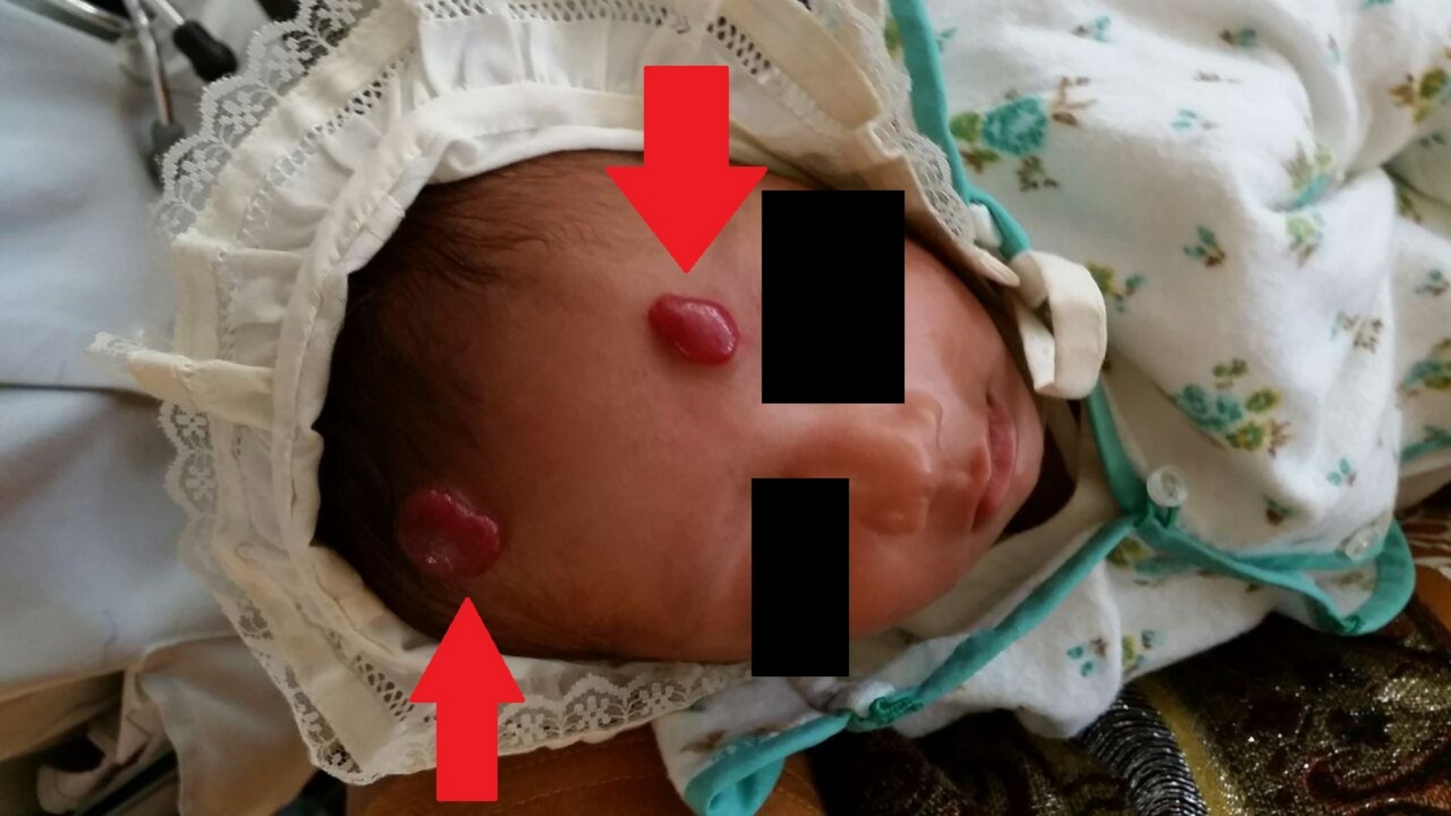
Red arrows showing the two hemangiomas found at birth on the newborn.

**Figure 3:**
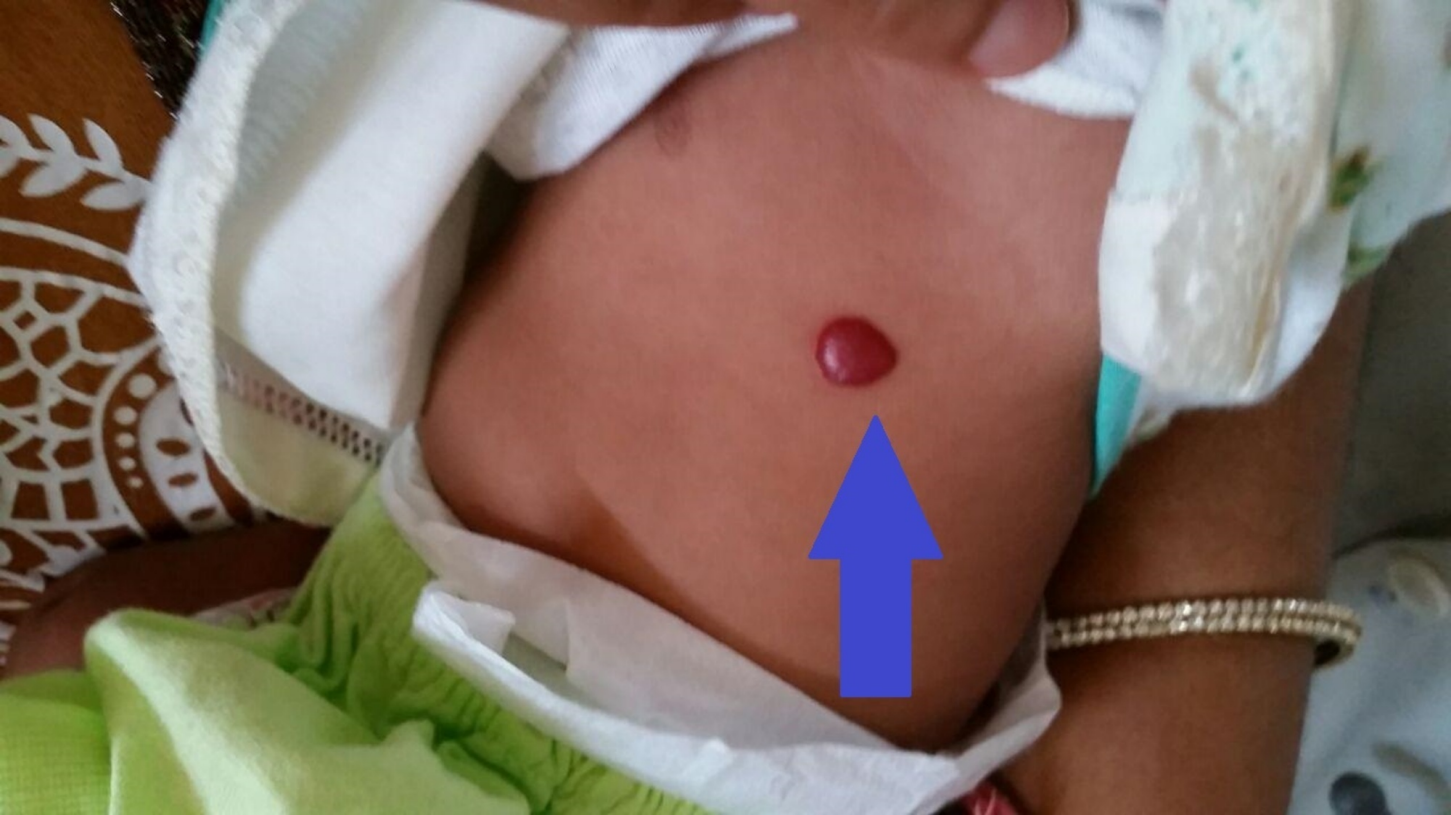
Blue arrow showing one hemangioma observed on the abdomen of the newborn.

On physical exam, the child was alert and active. She was not jaundiced and no pallor was noted on the extremities. Her vitals were all within the normal range and the birth weight and changes in weight corresponded properly. Two masses measuring 26 mm by 19 mm and 19 mm by 17 mm were observed on the forehead and the scalp (Figures 1-2). One smaller mass was seen on the abdomen, measuring 11 mm by 10 mm (Figure 3). All three masses were red and non-hemorrhagic. The parents reported that the size of the masses did not change since birth. No similar family history was found and both parents were healthy. Any hepatosplenomegaly was not observed on palpation nor were any other cutaneous lesions detected. She did not present with any other systemic abnormalities. An ultrasound was performed, and it revealed a normal liver, spleen, and kidneys with no masses or lesions. A Doppler examination also showed a fast-flow vascular lesion, as reported by many other articles [3]. The full blood count showed normal levels of white blood cells, platelets, hematocrit, and red blood cells.

The diagnosis of congenital hemangioma was confirmed and the family was properly educated about the condition as well as possible complications. A wait and watch approach was adopted and the child was scheduled for regular visits at three months. The family was asked to properly monitor the size of the masses and informed that appropriate surgical treatment would be provided if the masses fail to regress or if they continue to grow.

## Discussion

Congenital hemangiomas are rare vascular tumors that present as fully grown masses at birth. They usually appear as solitary or multiple soft tissue masses with coarse telangiectasia [4]. Since not all CHs regress, a proper follow-up is required to prevent any ulceration or bleeding from the tumor. NICHs have been reported to grow with age. RICHs are self-resolving and no treatment is actually needed. It is vital to rule out other conditions presenting with similar symptoms, such as either infantile hemangioma, which is usually a rapid postnatal growth, or Kaposiformhemangioendothelioma, which typically presents on histology as spindle-shaped cells, and any other highly vascular malignant tumor, such as rhabdomyosarcoma, angiosarcoma, and dermatofibrosarcoma. Any rapidly growing mass that is firm on palpitation, shows signs of ulceration, and has an atypical appearance should be considered for biopsy to rule out any malignancy. Infants with uncomplicated RICH usually have a good prognosis while infants with severe bleeding and who develop heart failure are at risk for complications. The latter condition was presented in a review published by Weitz et al., whereby out of 17 patients with large CH and heart failure, four died in the first weeks of life despite appropriate care [5].

The patient has been compliant with her follow-ups and for the last six months, the three masses did not increase in size. No new symptoms have also been reported during the follow-ups. Surgical resection is usually considered for esthetic reasons when patients reach pre-school age [6].

## Conclusions

Congenital hemangiomas have a low incidence rate, and it is vital for every physician to properly diagnose this condition. While usually, most physicians prefer to adopt a wait-and-watch approach, a proper differential diagnosis to rule out other conditions with similar symptoms should be appropriately performed. For congenital hemangiomas that do not regress, surgical excision can be considered once the child is of pre-school age.
